# Heating up the Blunts: Prothrombin Activation, with Factor Va as an Obligate Cofactor, Is the Dominant Procoagulant Mechanism of Blunt-Nosed Viper Venoms (*Macrovipera* Species)

**DOI:** 10.3390/toxins17080398

**Published:** 2025-08-08

**Authors:** Patrick S. Champagne, Lorenzo Seneci, Bryan G. Fry

**Affiliations:** Adaptive Biotoxicology Lab, School of the Environment, University of Queensland, St. Lucia, QLD 4072, Australia; p.champagne@uq.edu.au (P.S.C.); l.seneci@student.uq.edu.au (L.S.)

**Keywords:** venom, evolution, coagulopathy, factor activation

## Abstract

Venoms of the Palearctic vipers in the *Macrovipera* genus cause severe procoagulant clinical effects, yet the precise molecular targets remain incompletely defined. To fill this toxicological knowledge gap, we tested five *Macrovipera* venoms—*M. lebetina cernovi*, *M. l. obtusa*, *M. l. turanica* (Turkmenistan and Uzbekistan localities), and *M. schweizeri*—using plasma clotting assays, Factors VII, X, XI, and XII and prothrombin zymogen activation assays, and SDS-PAGE to visualise Factor V (FV) cleavage. All venoms induced extremely rapid clot formation (10.5–12.5 s) compared with the negative control (spontaneous clotting) of 334.6 ± 3.6 s) and the positive control (kaolin trigger) of 55.8 ± 1.9 s. Activation of FVII or FXI was negligible, whereas consistent FX activation and species-variable FXII activation, both moderate, were observed. Prothrombin remained inert in the absence of cofactors, but the presence of FV or FVa elicited potent thrombin generation. SDS-PAGE confirmed proteolytic conversion of the 330 kDa FV zymogen into the ~105 kDa heavy and ~80 kDa light chains of FVa by the venoms of all species. This data demonstrates that *Macrovipera* venoms rely on a dual enzyme strategy: (i) activation of FV to FVa by serine proteases and (ii) FVa-dependent prothrombin activation by metalloproteases. These results reveal that prothrombin activation is the dominant procoagulant pathway and overshadows the historically emphasised FX activation. This mechanism mirrors, yet is evolutionarily independent from, the FXa:FVa prothrombinase formation seen in Australian elapid venoms, highlighting convergent evolution of cofactor-hijacking strategies among snakes. The discovery of potent FVa-mediated prothrombin activation in *Macrovipera* challenges existing paradigms of viperid venom action, prompts re-evaluation of related genera (e.g., *Daboia*), and underpins the design of targeted antivenom and therapeutic interventions.

## 1. Introduction

Venom has evolved independently at least 100 times across the animal kingdom, spanning the full range of vertebrates and invertebrates [1,2,3,4]. Venom modes of action include the following: coagulotoxicity, which may be either anticoagulant through disruption of the clotting cascade to prevent clot formation or procoagulant through aberrant triggering of the cascade, such as to produce out of control clotting, leading to stroke or consumptive coagulopathy; cardiotoxicity, which is selective action upon cardiac or vascular smooth muscle cells; cytotoxicity, which is cell dysfunction and cell death/necrosis; myotoxicity, which is selective action upon skeletal muscle cells; and neurotoxicity, which is disruption or dysfunction of nerve impulse transmission [2,5,6,7]. These pathophysiological actions serve multiple ecological functions, including prey acquisition, defence, and intraspecific competition [3].

*Macrovipera*, known as blunt-nosed vipers, is a genus of large-bodied Palearctic vipers with procoagulant venoms that inhabit the semideserts and steppes of North Africa, the Near and Middle East, and the Cyclades Archipelago in the Aegean Sea: *M. lebetina cernovi* (Chernov Blunt-nosed Viper) is distributed across Northeast Iran, southern Turkmenistan, parts of northern Afghanistan and Pakistan (Kashmir); *M. l. lebetina* (Levantine Blunt-nosed Viper) is found in Cyprus; *M. l. obtusa* (Dagestan Blunt-nosed Viper) is distributed across the Caucasus region, including Dagestan and extending through central Turkey to northern Pakistan (Kashmir); *M. l. transmediterranea* (African Blunt-nosed Viper) is found in Algeria and Tunisia; *M. l. turanica* (Turan Blunt-nosed Viper) is distributed across the more eastern Central Asian regions, including parts of Turkmenistan, Uzbekistan, Tajikistan, Kyrgyzstan, and Afghanistan; and *Macrovipera schweizeri* (Milos Blunt-nosed Viper) is an endangered species restricted to the western side of the Cyclades archipelago, endemic to the islands of Kimolos, Milos, Polýaigos, and Sifnos [8,9]. The taxonomic status of *Macrovipera schweizeri* is the subject of ongoing debate; however, authors of numerous papers continue to consider it as a distinct species [8,10]. Similarly, there is debate as to whether the Levantine Blunt-nosed Viper subspecies should be referred to as *Macrovipera lebetina* or *Macrovipera lebetinus*. We follow Avella et al. 2025 in continuing the use of *Macrovipera lebetina* to avoid confusion [8], which is linked to a vast amount of literature, including almost all toxinological literature, while the use of *Macrovipera lebetinus* is restricted only to a small number of citations. Briefly, Frétey (2019) infers that Linnaeus (1758) used “lebetinus” not as an adjective, but rather as a noun, and that therefore there is no need for gender agreement between genus and species names [11]. However, Linnaeus never actually stated this. Furthermore, the Levantine viper has been named *M. lebetina* for 100+ years, so changing to *M. lebetinus* goes against the main aim of the Code of promoting stability in zoological nomenclature

The geographic separation and ecological adaptation to different biogeographic regions of the *M. lebetina* complex (Caucasian vs. Central Asian), as well as the isolation of the island-dwelling *M. schweizeri*, may have resulted in venom variations between these species/subspecies [8]. Understanding these potential differences in coagulation factor activation is crucial for both clinical and evolutionary perspectives, as variations in procoagulant activity could reflect adaptive responses to different prey types and predation pressures across distinct biogeographic zones [12]. This genus specifically is responsible for a number of bites of medical significance throughout its extensive distribution [13]. Despite the medical significance of *Macrovipera* envenomation and the established role of coagulation disorders in their pathophysiology, comprehensive comparative studies examining multiple factor activation patterns across the genus remain limited. Historically, research into this genus’ procoagulant mechanism of action has focused on the ability to activate the zymogen Factor X (FX) into the active enzyme form Factor Xa (FXa) [8,14,15,16,17,18,19,20]. Only a single study has examined the ability to activate clotting factors other than FX, which showed that Factor XII and VII were also activated, with a rank order of FX > FXII > FVII [15]. *Macrovipera* venoms, however, lack direct thrombin-like/pseudo-procoagulant direct action upon fibrinogen [21].

The objectives of this study were to investigate and compare the activation of coagulation factors V, VII, X, XI, and XII and prothrombin by venoms from different *Macrovipera* taxa. By examining this comprehensive panel of coagulation factors across geographically distinct populations, this research aims to elucidate potential subspecies-specific variations in procoagulant activity and contribute to our understanding of the evolutionary diversification of venom composition within the *Macrovipera* genus.

## 2. Results and Discussion

**Figure 1:**
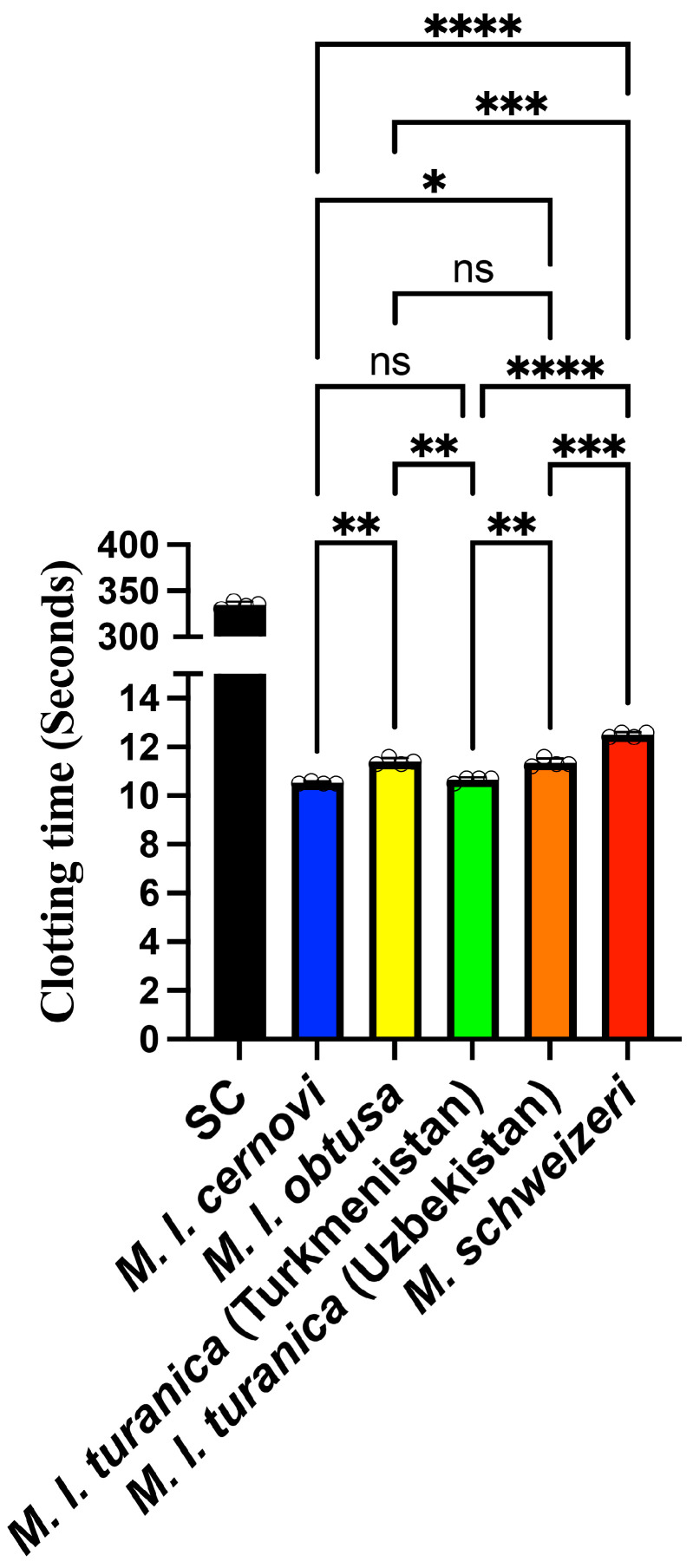
Venom-induced clotting times in human plasma. All values are N = 4 ± standard deviation. *SC* = the negative control (spontaneous clotting), the time to which recalcified plasma will clot without the addition of external triggers. All values are N = 4, with the mean and standard deviation. Statistics are Brown–Forsythe and Welch ANOVA tests with post hoc Dunnett T3 multiple comparisons. **** = *p* < 0.0001, *** = *p* < 0.001, ** = *p* < 0.01, * = p < 0.05, and ns (non-significant) = *p* ≥ 0.05.

The plasma clotting negative control (spontaneous clotting) was 334.6 ± 3.6 s, and the positive control (kaolin trigger) was 55.75 ± 1.9 s. All *Macrovipera* venoms dramatically shortened the clotting time relative to the spontaneous clotting control (Figure 1): *M. l. cernovi*: 10.53 ± 0.05 s; *M. l. turanica* (Turkmenistan): 10.65 ± 0.10 s; *M. l. turanica* (Uzbekistan): 11.35 ± 0.17 s; *M. l. obtusa*: 11.40 ± 0.14 s; and *M. schweizeri:* 12.50 ± 0.12 s.

**Figure 2:**
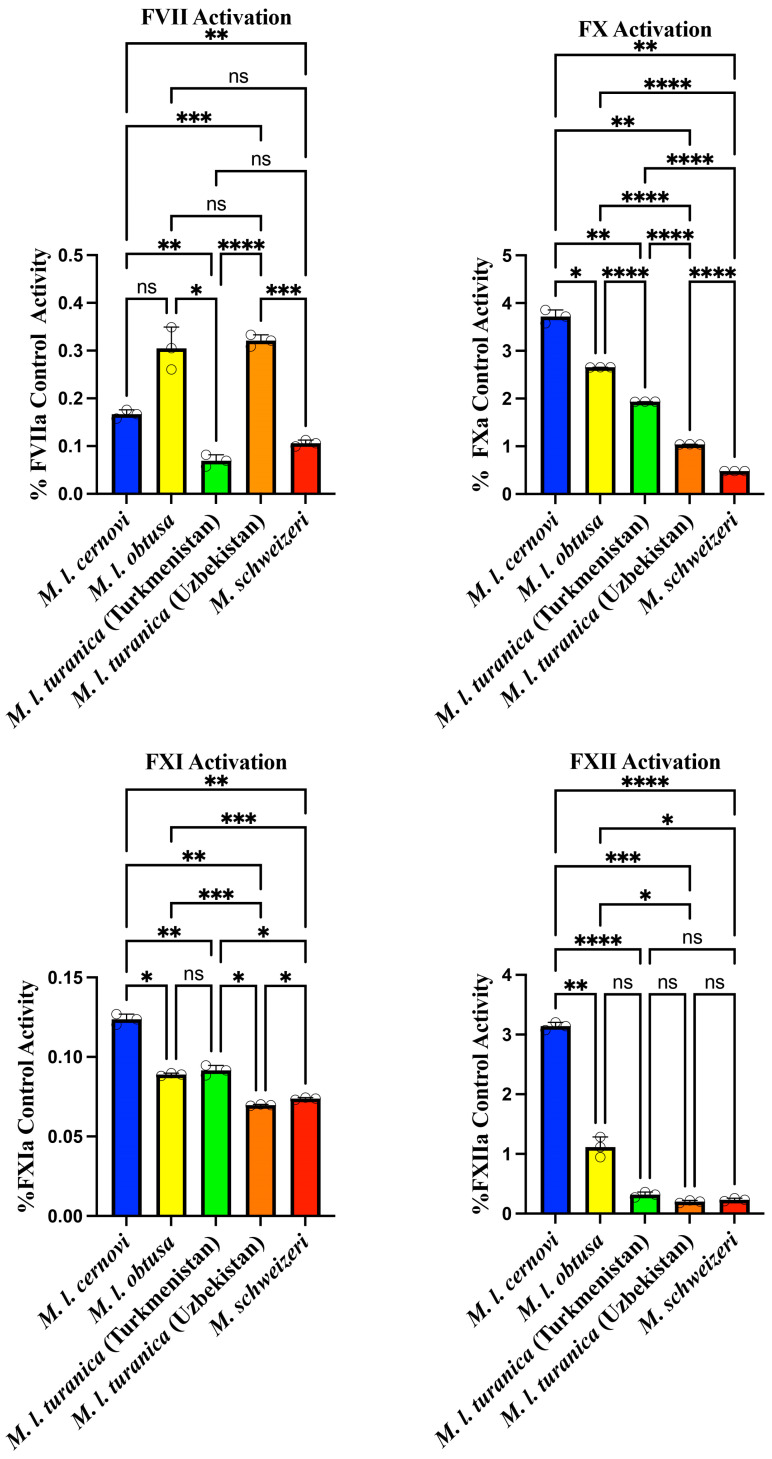
Coagulation factor activation by *Macrovipera sp.* venoms measured using fluorogenic substrate assays. All values are N = 3 ± standard deviation. Note, the y-axes are not uniformly scaled in order to facilitate visualisation of the data. The negative controls of zymogens by themselves produced no reaction. All values are N = 3, with the mean and standard deviation. Statistics are Brown–Forsythe and Welch ANOVA tests with post hoc Dunnett T3 multiple comparisons. **** = *p* < 0.0001, *** = *p* < 0.001, ** = *p* < 0.01, * = p < 0.05, and ns (non-significant) = *p* ≥ 0.05.

In order to elucidate the underlying biochemistry responsible for these procoagulant traits, we next ascertained cleavage of different clotting factor zymogens (Factors VII, X, XI, XII, and prothrombin). The FVII and FXI activation assay results reveal that all tested *Macrovipera* sp. demonstrate very low intrinsic capacity to activate these two factors (Figure 2). While there were variations between them, these differences were not biologically relevant considering the very low levels of activity. The results suggest that direct FVII of FXI activation is not a primary mechanism employed by these venoms for disrupting the coagulation cascade. 

FX and FXII activation results show markedly different patterns compared to the FVII data, with all *Macrovipera* sp. demonstrating moderate FX activation capacity (Figure 2), which is consistent with *Macrovipera* venoms having been previously characterised as activating this factor [8,14,15,16,17,18,19,20]. There were notable variations between the venoms: *M. l. cernovi* exhibited the highest activation potential at 3.72 ± 0.14% followed by *M. l. obtusa* at 2.65 ± 0.00396%. *M. l. turanica* (Turkmenistan) showed moderate activation capacity at 1.93 ± 0.00121%, while *M. l. turanica* (Uzbekistan) demonstrated lower activation at 1.04 ± 0.000539%. *Macrovipera schweizeri* displayed notably weaker FX activation capacity at 0.481 ± 0.000493%. As with FX, FXII activation results demonstrate species-specific variation in activation capacity, with values ranging from 0.18% to 3.2% (Figure 2). *Macrovipera l. cernovi* exhibited the highest FXII activation potential at 3.14 ± 0.0652%, representing a substantially greater potency than all other tested samples. *M. l. obtusa* showed moderate activation at 1.12 ± 0.169%, while the remaining three venoms displayed much lower activation capacities: *Macrovipera l. turanica* (Turkmenistan) at 0.318 ± 0.0450%, *M. schweizeri* at 0.233 ± 0.0250%, and *M. l. turanica* (Uzbekistan) showing the lowest activation at 0.201 ± 0.0219%. The dramatic difference between *M. l cernovi* and the other species is particularly striking, with *M. l cernovi* showing more than double the activation capacity of *M. l. obtusa* and over ten times that of the weakest activators. These results suggest that Factor XII activation may be a more significant mechanism for *M. l cernovi* venom compared to the other *Macrovipera sp.* tested. The differences for *M. l. obtusa* in this study relative to prior work, which showed more significant FXII activity, points to possible regional variation in venom action within this subspecies [15].

**Figure 3:**
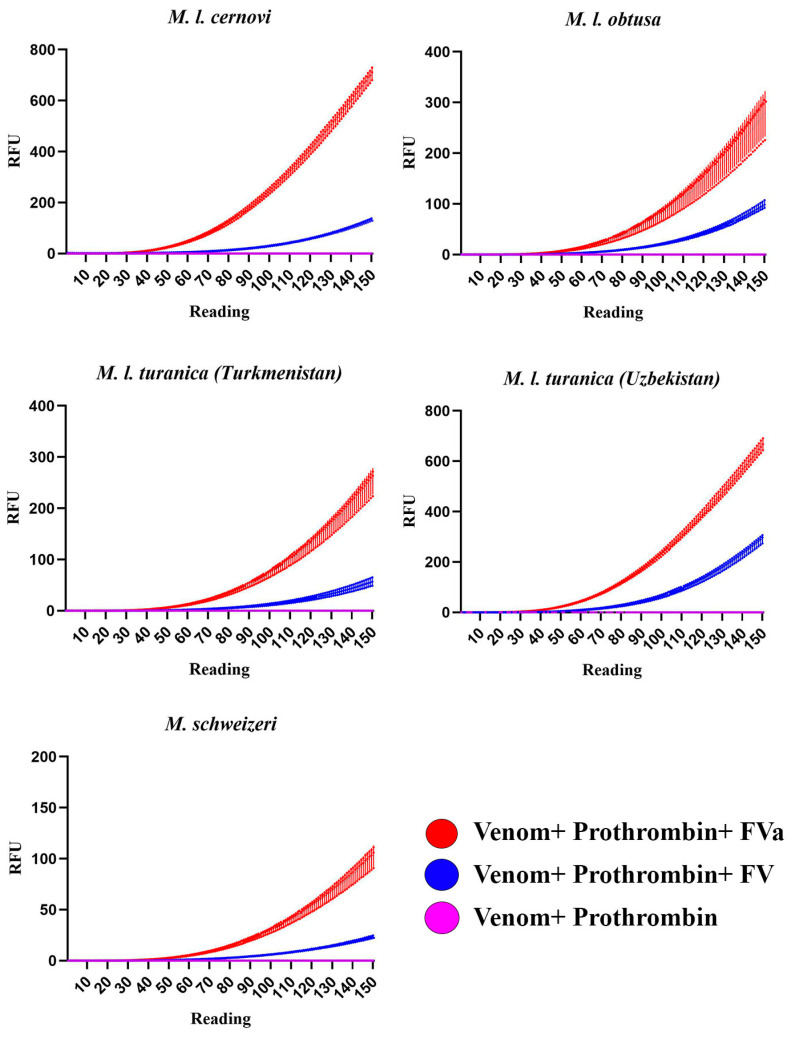
Time–course analysis of prothrombin activation by *Macrovipera* venoms under different cofactor conditions. Fluorometric kinetic traces showing thrombin generation over time (readings) for three experimental conditions: venom with prothrombin alone (purple), venom with prothrombin plus Factor V (blue), and venom with prothrombin plus Factor Va (red), (blue). *RFU* = Relative Fluorescence Units. Note, the y-axes are not uniformly scaled in order to facilitate visualisation of the data. All values are N = 3, with the mean and standard deviation. Readings are 30 s apart, for a total run time of 75 min.

**Figure 4:**
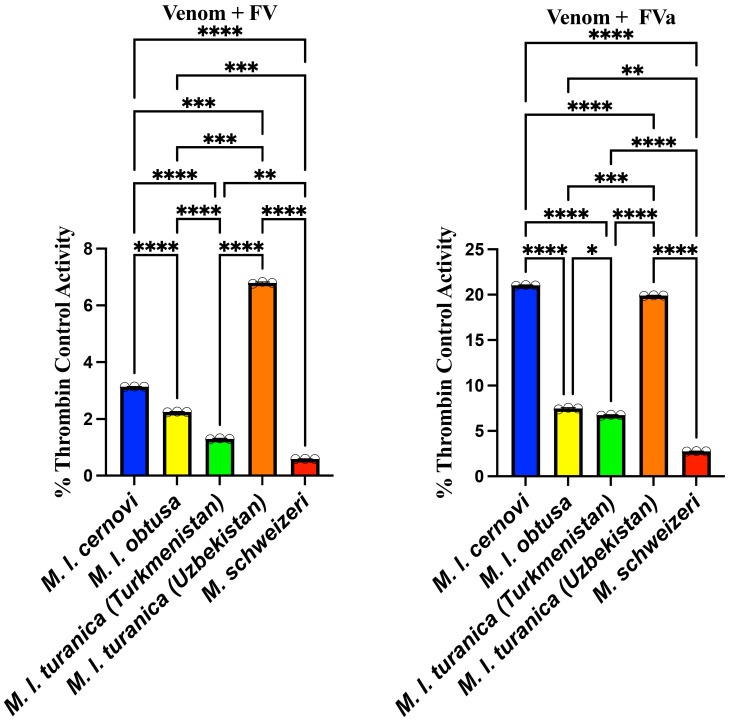
Prothrombin activation by *Macrovipera* venoms in the presence of Factor V cofactors. The left panel shows prothrombin activation capacity with Factor V (FV) as a cofactor, while the right panel shows activation with activated Factor Va (FVa) as cofactor. The results are expressed as a percentage of thrombin control activity. Note, the y-axes are not uniformly scaled in order to facilitate visualisation of the data. All values are N = 3, with the mean and standard deviation. Statistics are Brown–Forsythe and Welch ANOVA tests with post hoc Dunnett T3 multiple comparisons. **** = *p* < 0.0001, *** = *p* < 0.001, ** = *p* < 0.01, * = p < 0.05, and ns (non-significant) = *p* ≥ 0.05.

All of the venoms displayed trivial levels of prothrombin activation when incubated with the zymogen alone, ranging from 0.0115 ± 0.000332% for *M. l cernovi* to 0.00179 ± 0.000165% for *M. l. turanica* (Turkmenistan). Based on a recent report of the South American pit viper *Bothrocophias campbelli* also being inactive when incubated with just prothrombin, but potently activating it when the cofactor Factor Va (FVa) was added to the experimental conditions [22], the *Macrovipera* prothrombin activation reactions were rerun under two additional conditions: with Factor V (FV) to test for the venom’s ability to activate FV into FVa and generate its own bioavailable cofactors, and with FVa to test for the ability to use endogenous FVa produced via FX activation. These tests revealed that all of the *Macrovipera* venoms were potent activators of prothrombin when either FV or FVa was present (Figure 3 and Figure 4), demonstrating that not only do these venoms require FVa as a cofactor for prothrombin activation but that they are also able to activate FV into FVa to produce this cofactor. Prothrombin activation proceeded at a faster rate for the FVa reactions than the FV reactions, which is consistent with more biochemical steps being involved in the latter reaction, since the venoms are undertaking the catalytic reactions to convert FV into FVa. All venoms showed a marked increase in the relative rate of reaction for prothrombin activation for FV versus FVa: *M. l. cernovi* increasing from 3.13 ± 0.00680% with FV to 21.01 ± 0.0411% with FVa, a 671% change in relative activity; *M. l. obtusa* increasing from 2.25 ± 0.0107% with FV to 7.48 ± 0.0932% with FVa, a 332% change in relative activity; *M. l. turanica* (Turkmenistan) increasing from 1.3 ± 0.0151% with FV to 6.76 ± 0.0479% with FVa, a 520% change in relative activity; *M. l. turanica* (Uzbekistan) increasing from 6.8 ± 0.0317% with FV to 19.9 ± 0.0326% with FVa, of 293% change in relative activity; and *M. schweizeri* increasing from 0.586 ± 0.00123% with FV to 2.76 ± 0.0187% with FVa, a 471% change of in relative activity. The patterns between venom + FV and venom + FVa were not identical due to the assays testing for different aspects. The FV assay tested for the ability to convert FV into FVa and also the ability to use FVa as a cofactor. The FVa assay, however, simply tested for the ability to use FVa as a cofactor. Therefore, the results would not be expected to be the same, as it would be expected that the venoms differed in their ability to convert FV into FVa while also differing in their ability to use FVa as a cofactor. The two controls (FV incubated with prothrombin and FVa incubated with prothrombin) displayed no activity, underscoring that it is a cofactor-mediated venom activity, not an activity driven by FV or FVa.

**Figure 5:**
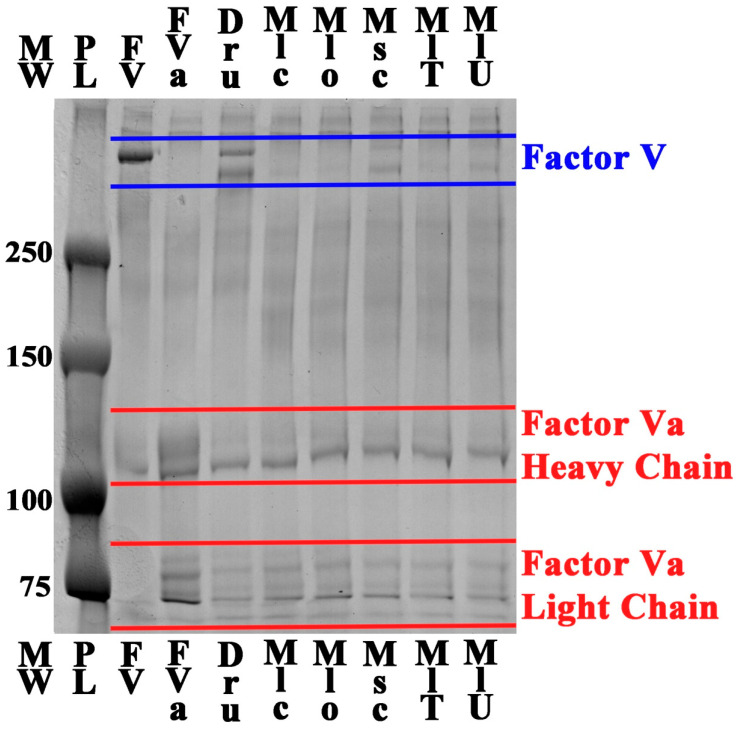
SDS-PAGE analysis of Factor V activation by *Macrovipera* venoms. Lanes show molecular weight markers (MW), protein ladders (PL), the Factor V control lane (FV), the Factor Va control lane (FVa), the *Daboia russelli* control lane (Dru), and venom-treated samples for *M. lebetina cernovi* (Mlc), *M. lebetina obtusa* (Mlo), *M. schweizeri* (Msc), *M. l. turanica* Turkmenistan locality (MlT), and *M. l. turanica* Uzbekistan locality (MlU). Blue lines indicate intact Factor V (~330 kDa), while red lines highlight Factor Va heavy chain (~105 kDa) and light chain (~80 kDa) fragments, demonstrating species-specific differences in Factor V cleavage and activation capacity. In order to visualise the high-molecular-weight proteins that make up FV and FVa, the gel was run until the 75 kDa marker was just above the bottom edge.

The ability of the venoms to convert FV into FVa was confirmed through the use of 1D SDS page gels, which monitored the physical cleavage of FV into the heavy and light chains of the activated form (Figure 5). All tested venom samples demonstrated clear evidence of Factor V proteolytic cleavage, as shown by the characteristic band pattern changes consistent with the conversion of the single-chain Factor V zymogen (330 kDa) to the activated Factor Va heterodimer comprising heavy chain (~105 kDa) and light chain (~80 kDa) subunits. The positive control (*Daboia russelli* venom) confirmed the expected Factor V activation pattern, validating the experimental conditions and demonstrating the characteristic proteolytic signature of known Factor V activators. These band patterns are consistent with previous work, which shows that *Daboia* and *Macrovipera* activate FV by cleaving it at the Arg1545-Ser1546 bond [23,24,25]. This is the site important for the formation of the prothrombinase complex necessary for thrombin generation in hemostasis. The negative control (Factor V alone) showed no spontaneous activation, confirming that observed band changes were specifically due to venom-mediated proteolysis rather than thermal or chemical degradation during the assay conditions.

Previously, it has been shown that *Bothrocophias campbelli, Bothrops atrox, Daboia russelii*, *Macrovipera lebetina*, and *Vipera ursini* venoms are able to cleave Factor V into the active form Factor Va [22,23,24,25,26,27,28]. The toxin-type responsible has been shown to be a kallikrein-scaffold serine protease in the true viper *Daboia*, *Macrovipera*, and *Vipera* venoms and is presumably the same for the pit viper genera, but this remains to be experimentally confirmed. However, only venoms in the pit viper genus *Bothrocophias* have been previously tested for their ability to utilise Factor Va to activate prothrombin [22]. As such, the results of this study not only contribute to the body of knowledge on venom evolution in vipers but also provide new information regarding the dominant mode of action on the blood chemistry. Most notably, FVa-mediated activation of prothrombin is the dominant coagulotoxic activity in this venom, far exceeding the FX activation thought previously to be the major pathophysiological mechanism. Future work should re-examine the mechanisms of action in other genera, such as *Daboia* (close relatives of *Macrovipera* and of high medical significance), to ascertain if FVa-mediated activation of prothrombin is also a major overlooked toxic action.

The use of FVa as a cofactor for zymogen activation is a trait that has evolved convergently on at least two occasions within snakes. At least once within the viperid snake venoms, which use metalloprotease enzymes for zymogen activation, and again in the Australian elapid snakes, which use a weaponised form of the blood clotting enzyme Factor Xa instead of metalloproteinases [29,30,31,32,33,34,35,36,37,38,39,40,41,42,43,44,45,46,47]. In these elapid snakes, the use of FXa with FVa as a cofactor evolved to activate FVII as the basal trait within this clade, with prothrombin activation observed only for some derived species [29]. In these snakes, endogenous FVa is used by all lineages except for the species within the *Oxyuranus/Pseudonaja* clade, which have secondarily evolved to express FVa within the venom, thereby eliminating the need to obtain endogenous FVa for activity [48].

In contrast to the Australian elapids, which replicate the endogenous prothrombinase complex through the use of the FXa blood clotting enzyme as a toxin, in viperid snake venoms a functional prothrombinase complex is formed through the neofunctionalisation of metalloprotease enzymes into forming functional prothrombinase complexes with Factor Va, essentially mimicking Factor Xa’s mechanism rather than bypassing the natural coagulation cascade.

This cofactor dependence is demonstrated by the complete absence of prothrombin activation without Factor V/Va present, indicating an obligate requirement for the cofactor, underscoring the critical role of FVa in binding to prothrombin in order to cleave it into the active form thrombin. This mechanism represents an elegant evolutionary strategy—rather than developing completely independent procoagulant enzymes, viperid snake venoms have evolved to hijack and amplify the existing prothrombinase pathway. Notably, *Macrovipera* venoms also demonstrate Factor V-activating activity alongside their prothrombinase-like functions, creating a highly efficient positive feedback loop. The venom simultaneously generates Factor Va cofactor and utilises it for enhanced prothrombin conversion, representing a sophisticated dual-mechanism approach to coagulation activation.

Despite producing highly similar times for rapid clot formation of human plasma, the underlying biochemistry varied between the venoms. The differential activation patterns observed across the various coagulation factors reveal distinct mechanistic strategies employed by *Macrovipera* venoms, with particularly intriguing differences between the *M. lebetina* complex species and *M. schweizeri*. All species demonstrated minimal capacity for direct Factor VII and XI activation, suggesting that these pathways are not primary targets. The species-specific variation in Factor XII activation, particularly the higher potency of *M. l cernovi*, suggests potential evolutionary divergence in contact pathway exploitation. In contrast, the high levels of Factor X activation in all species indicate that this may represent a more significant mechanism for initiating coagulation disruption, as do the extremely high levels of prothrombin activation, as the dominant type of factor activation for all species suggests that this has a central role in prey subjugation. Moreover, *M. schweizeri* venom was notably slower than the *M. lebetina* subspecies’ venoms in activating all of the zymogens tested, despite clotting human plasma only marginally slower (but still extremely potent). This suggests that the venom of this species may be acting elsewhere in the clotting cascade, such as activation of the coagulation enzyme Factor IX, which could not be tested in this study. Future work should also investigate if FVa is used as a cofactor for the activation of clotting factors other than just prothrombin, which may also explain the discrepancy noted for *M. schweizeri* clotting time versus factor activation profile.

These findings indicate that *Macrovipera* venoms employ factor-specific and species-specific approaches to coagulotoxicity, with FV, FX, and prothrombin activation appearing as the most conserved mechanisms across the genus, while Factor XII activation represents a more specialised mechanism that varies considerably between species. This suggests that organismal radiation and separation have led to divergence not only in morphological and genetic characteristics but also in venom composition and function, suggesting that evolutionary pressures have shaped distinct coagulation-targeting strategies.

## 3. Materials and Methods

### 3.1. Venom Sources and Preparation

Lyophilised venoms were obtained from the licensed biosupply company Latoxan: *Macrovipera lebetina cernovi* Turkmenistan locality (catalogue #L1144), *M. l. obtusa* from Azerbaijan (catalogue #L1126), *M. l. turanica* from Turkmenistan (catalogue #L1128), *M. l. turanica* Uzbekistan and Turkmenistan localities (catalogue #L1128), and *M. schweizeri* Greece locality (catalogue #L1127). Venoms were received under University of Queensland Animal Ethics Approval (15 March 2021/AE000075), and the work was approved by UQ Biosafety Committee Approval #IBC/134B/SBS/2015 (14 April 2023). Venoms were stored at 80 °C before reconstitution. During the study, 1 mg of each venom was transferred into a 1.5 mL Eppendorf tube under sterile conditions. Subsequently, ddH_2_O (double-distilled water) was added to the sample before vortexing for 5 s and centrifugation (4 °C, 14,000 RCF; 10 min). The supernatant was then transferred to another 1.5 mL Eppendorf tube, and the protein concentration was determined in triplicate on a Nanodrop 2000 spectrophotometer in the 260/280 nm absorbance range (Thermo Fisher Scientific, Sydney, Australia), with light vortexing between replicates. Lastly, glycerol was added to reach a final venom concentration of 1 mg/mL. The reconstituted venom samples were aliquoted into 200 μL tubes and stored at −80 °C until used.

### 3.2. Stago STA-R Max Coagulation Assays

All human plasma work was conducted under University of Queensland Biosafety Committee Approval #IBC/149B/SBS/2016 (20 September 2023), UQ Human Ethics Approval #2016000256 (9 May 2024), and Australian Red Cross Research Agreement #16- 04QLD-10 (2 February 2025). Clotting time of human plasma by *Macrovipera* venoms was assessed using coagulation assays performed on a Stago STA-R Max haemostasis analyser (Stago, Forest Hill, VIC, Australia), following validated protocols [15]. Frozen citrated human plasma (3.2%) was thawed and equilibrated at 37 °C in a water bath for 5 min. Venom from a 1 mg/mL glycerol stock was diluted with Owren–Koller (OK) buffer (Stago #00360, Stago, Australia) to a working concentration of 100 μg/mL. The STA-R Max added 50 μL of diluted venom to a mixture containing 50 μL CaCl_2_ (25 mM stock solution Stago #00367, Stago, Australia), 50 μL phospholipid (Stago #00597, Stago, Australia), and 25 μL OK buffer. Calcium and phospholipid were included to mimic physiological conditions as they are coagulation cofactors. This mixture was incubated at 37 °C for 120 s to ensure complete mixing, followed by the addition of 75 μL of human plasma, resulting in a final venom concentration of 20 μg/mL. Clotting time was recorded upon plasma addition. Each venom concentration was tested in quadruplicate. The spontaneous clotting time of human plasma was used as a negative control by replacing venom with 50% ddH_2_O–glycerol, whereas the positive control consisted of an activated partial thromboplastin time (APTT) with the addition of 50 μL kaolin (Stago Cat# 00597, Stago, Australia).

### 3.3. Fluoroskan Assays for FVII, FX, FXI, FXII, and Prothrombin Activation

Zymogen activation studies were undertaken in a Fluoroskan Ascent™ (Thermo Scientific, Vantaa, Finland) with 384-well plates (black, lot #1171125, Nunc™ Thermo Scientific, Rochester, NY, USA) using a slightly modified version of already validated protocols from our lab [10]. Plates for FVII, FX, FXI, and FXII were run with 0.1 μg venom + 1 μg zymogen/enzyme per well, whereas prothrombin activation was measured at 0.01 μg venom + 0.1 μg zymogen/enzyme per well due to the extremely high affinity of thrombin with the fluorometric substrate.

More specifically, plates were run at 37 °C and shaken for 3 s before each measurement to obtain uniform mixing. Fluorescence levels were measured for 150 readings using excitation and emission wavelengths of 390 nm and 460 nm, respectively. Cross-contamination between runs was prevented by cleaning the Fluoroskan interior with 95% ethanol, followed by 100% ddH_2_O, and finally priming with the reaction substrate solution containing Fluorogenic Peptide Substrate ES011 (Boc-Val-Pro-Arg-AMC; R&D Systems, catalog #ES011, Minneapolis, MN, USA) diluted in 5 mM CaCl_2_ + OK buffer in a 500:1 ratio (250:1 for prothrombin activation). The reaction conditions for each plate are reported as follows:

#### 3.3.1. Activation of FVII, FX, FXI, or FXII Plate Setup [15]

##### Blank Control Wells

- 10 μL phospholipid (Stago #00597)
- 20 μL of OK buffer

##### Zymogen Control Wells

- 10 μL phospholipid (Stago #00597)
- 10 μL of 10 μg/mL of either FVII (Prolytix #HCVII-0030), FX (Prolytix #HCX-0050), FXI (Prolytix #HCXI-0150), FXII (Prolytix #HCXII-0155), or prothrombin (Prolytix # HCP-0010)
- 10 μL of OK buffer

##### Activated Enzyme Control Wells

- 10 μL phospholipid (Stago #00597)
- 10 μL of 10 μg/mL of either FVIIa (HCVIIA-0031), FXa (Prolytix # HCBXA-0061), FXIa (Prolytix # HCXIA-0160), FXIIa (Prolytix #HCXII-0155 activated with kaolin), or thrombin (Prolytix # HCT-0020)
- 10 μL of OK buffer

##### Venom Control Wells

- 10 μL phospholipid (Stago #00597)
- 10 μL of 1 μg/mL venom
- 10 μL of OK buffer

##### Experimental Wells

- 10 μL phospholipid (Stago #00597)
- 10 μL of 10 μg/mL of either FVII (Prolytix #HCVII-0030), FX (Prolytix #HCX-0050), FXI (Prolytix #HCXI-0150), FXII (Prolytix #HCXII-0155), or prothrombin (Prolytix # HCP-0010)
- 10 μL of 1 μg/mL venom

#### 3.3.2. Prothrombin Activation Plate Setup [29]

##### Blank Control Wells

- 10 μL phospholipid (Stago #00597)
- 20 μL OK buffer

##### Zymogen Control Wells

- 10 μL phospholipid (Stago #00597)
- 10 μL 1 μg/mL prothrombin (Prolytix HCP-0010)
- 10 μL OK buffer

##### Activated Enzyme Control Wells

- 10 μL phospholipid (Stago #00597)
- 10 μL 1 μg/mL thrombin (Prolytix HCT-0020)
- 10 μL OK buffer

##### Venom Control Wells

- 10 μL phospholipid (Stago #00597)
- 10 μL 0.1 μg/mL venom
- 10 μL OK buffer

##### Experimental Wells

- 10 μL phospholipid (Stago #00597)
- Prothrombin tested under three separate conditions:
  ○ 10 μL 1 μg/mL prothrombin (Prolytix HCP-0010)
  ○ 10 μL of a combined solution consisting of 1 μg/mL prothrombin (Prolytix HCP-0010) + 7 µg/mL Factor V (Prolytix #HCV-0100).
  ○ 10 μL of a combined solution consisting of 1 μg/mL prothrombin (Prolytix HCP-0010) + 4 µg/mL Factor Va (Prolytix #HCVA-0110).
- 10 μL 0.1 μg/mL venom

### 3.4. One-Dimensional Polyacrylamide Gel Electrophoresis (SDS-PAGE)

Venom samples (0.2 μg) were incubated at 37 °C for 30 min with 2 μg Factor V, 2 μL Ca^2+^, and 2 μL phospholipid diluted with 5 μL Owren–Koller buffer in a total volume of 7.5 μL. Following incubation, 7.5 μL of 2× Laemmli sample buffer (Lot number 1610737, Bio-Rad, Hercules, CA, USA) was added to each sample for a final volume of 15 μL. Control wells had the same conditions; however, in place of the 2 μL venom, a total of 7 μL OK buffer was added. In addition to the *Macrovipera* samples, a positive control containing *Daboia russelii* venom (a known Factor V activator) was included to assess the expected FV-venom reaction. Negative controls (FV only) and positive controls (FVa only) were included in each gel.

Samples were separated on 12% precast polyacrylamide gels (Bio-Rad, 12% Mini-PROTEAN^®^ TGX™ Precast Protein Gels, 15-well, 15 µL #4561046) The electrophoresis chamber was filled with 1× Tris/Glycine/SDS running buffer prepared by diluting 100 mL of 10× stock (Lot number 1610732, Bio-Rad, Hercules, CA, USA) in 900 mL ddH_2_O. A protein standard (Precision Dual Plus Protein™ Unstained Protein Standards, Strep-tagged recombinant, 1 mL, catalog #1610363, Bio-Rad, Hercules, CA, USA; molecular weight range 10–250 kDa) was used as a molecular weight reference.

Electrophoresis was performed in a PowerPac™ Basic Power Supply (Bio-Rad, Hercules, CA, USA) at 120 V until complete precipitation of the bands was observed. Following electrophoresis, gels were rinsed three times for 5 min each with ddH_2_O and then stained with 50 mL of 1 g/L Bio-Safe™ Coomassie Stain (#1610786, Bio-Rad, Hercules, CA, USA) for 1 h. Gels were subsequently destained in 50 mL ddH_2_O for 30 min.

Gel imaging was performed using a GS-900™ Calibrated Densitometer (Bio-Rad, Hercules, CA, USA) to observe venom action on Factor V. Automatic calibration was performed prior to each scan.

### 3.5. Statistics

Blood clotting assays were performed in quadruplicate (*n* = 4) and fluorometry runs in triplicate (*n* = 3). Prior to analysis, background fluorescence was corrected by subtracting “venom without zymogen” values from “venom with zymogen” values to eliminate non-specific substrate cleavage by venom components. The corrected values were then normalised as a percentage of the positive control (activated factors) using Excel, and statistical analysis of the area under the curve (AUC) was performed using GraphPad PRISM 10.4.1 (GraphPad Prism Inc., La Jolla, CA, USA).

## Data Availability

The original contributions presented in this study are included in the article. Further inquiries can be directed to the corresponding author.
